# Testicular Torsion: Successful Management of a Late-Diagnosed Case

**DOI:** 10.7759/cureus.16845

**Published:** 2021-08-02

**Authors:** Raghubir Bhardwaj, Somen Chakravarthy, Soumya Misra

**Affiliations:** 1 Urology, Tata Main Hospital, Jamshedpur, IND; 2 Radiology, Tata Main Hospital, Jamshedpur, IND; 3 Surgery, Tata Main Hospital, Jamshedpur, IND

**Keywords:** testis, torsion, exploration, orchidopexy, late-diagnosed

## Abstract

Testicular torsion is an emergency. It usually occurs spontaneously, without an apparent cause but has been associated with anatomical, traumatic, and environmental factors. In the case of the acute scrotum, a high degree of clinical suspicion is the most important factor in early diagnosis. Scrotal Doppler helps to confirm the diagnosis of testicular torsion. Prompt recognition and treatment are critical for testicular viability. Surgical intervention, even in late-diagnosed selected cases may yield desirable results. We report a case of a 16-year-old boy who came to the urology outpatient department (OPD) with a history of scrotal pain for approximately 12 hours.

## Introduction

For males of the age 25 years and younger, testis torsion is more than three times more common than testis cancer. It has an estimated incidence of 4.5 cases per 100,000 per year [1,2]. It is common among young males presenting with scrotal pain of sudden onset [3-5]. Testicular torsion occurs when the testis twists around the spermatic cord, resulting in compromised blood flow to the testis [6]. The degree of torsion and duration of symptoms are prognostic factors of testicular viability [7]. The treatment of choice is early surgical management. We report a case of a young boy aged 16 years who came to the urology outpatient department (OPD) with a history of scrotal pain for approximately 12 hours.

## Case presentation

**Figure 1:**
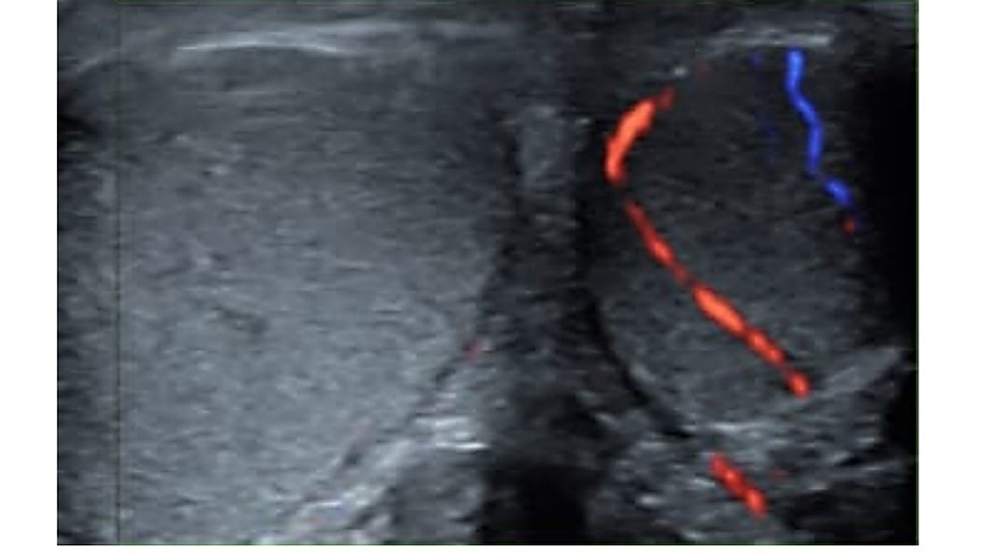
Scrotal Doppler shows no blood flow in the right testis

A young boy aged 16 years presented to the urology OPD of the hospital with a history of right testicular pain for more than 12 hours. There was associated waist pain but no history of trauma, fever, nausea, or vomiting. He was voiding well. On physical examination, the right testis was larger in volume as compared to the left, high riding, and tender to touch. Prehn’s sign (pain relief on lifting the affected testicle) was negative. Cremasteric reflex was absent on the affected side. Testicular Workup for Ischemia and Suspected Torsion (TWIST) score was 6. The clinical diagnosis of the right testicular torsion was made. The radiologist was informed over the phone regarding the need to do an urgent scrotal Doppler to confirm the diagnosis. At the same time, the anesthesiologist and emergency operation theatre (OT) team were also informed. Scrotal Doppler showed enlarged right testis with no blood flow; contralateral testis was normal (Figure 1). 

**Figure 2:**
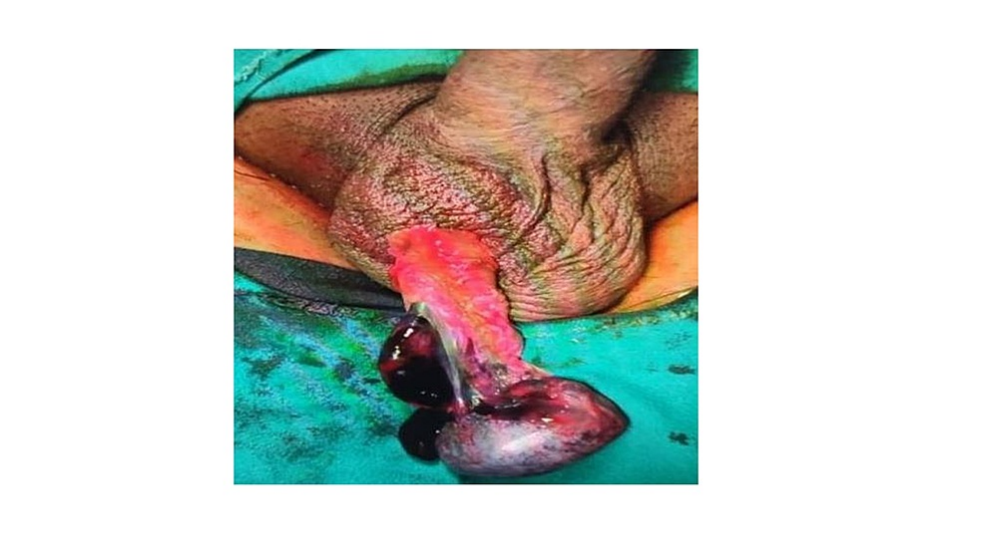
Testicular torsion - intraoperative picture

The patient was taken up for emergency scrotal exploration. A right-sided longitudinal scrotal incision was given, wound opened, and right testis delivered. It was oedematous and dark in color testicular torsion was identified and relieved (Figure 2). Testis was kept in the warm saline-soaked sponge and high flow oxygen was given to the patient to improve testicular perfusion. Colour change was noted after about 10-15 minutes. So, it was decided to salvage the affected testis, orchidopexy was done using a non-absorbable suture. Contralateral orchidopexy was performed to avoid torsion as a bell-clapper deformity that increases the risk of torsion is bilateral in up to 80% of cases. The patient did well after surgery. He was discharged on the third postoperative day with the advice to come for follow-up in urology OPD after one week. A follow-up examination showed good recovery. Scrotal Doppler at one month and three months showed bilateral normal testicular perfusion and echotexture.

## Discussion

Testicular torsion is not an uncommon urologic emergency. It represents approximately 25% of cases of acute scrotum presenting in an emergency room. The exact cause of testicular torsion is not well understood but certain anatomical factors like failure of normal posterior anchoring of gubernaculum, epididymis and, testis (bell-clapper deformity), horizontal lie, and mal-descended testis increase the possibility of testicular torsion [8,9]. It may follow trauma. The high index of suspicion helps in early diagnosis and prompt management and thus increases testicular salvage. Proper physical examination plays an important role in diagnosing testicular torsion as it is the most important diagnosis to rule out when a patient presents with testicular pain. Scrotal Doppler not only helps to confirm the clinical diagnosis but also predicts testicular viability based on parenchymal echotexture of the affected testis [10]. Delay in diagnosis and further management will affect the outcome.

One scoring system that can aid physicians in diagnosis is Testicular Workup for Ischemia and Suspected Torsion (TWIST). This scoring system was developed to diagnose testicular torsion on clinical grounds, thus decreasing the need for ultrasound [11]. TWIST consists of the following history and physical examination parameters: testis swelling (2 points), hard testis (2 points), absent cremasteric reflex (1 point), nausea/vomiting (1 point), and high-riding testis (1 point). 

Multiple review studies have confirmed that a score of >5 has a positive predictive value (PPV) of 100% and a score of <2 has a negative predictive value (NPV) of 100%. This scoring was initially derived from the pediatric population. It may still be helpful to the physician to make a quick diagnosis, especially when the USG facility is not available.

Filho et al. reported a correlation in time and degree of torsion in the orchidopexy group in 117 patients [12]. The viability of affected testis is determined by the degree of twisting and duration of symptoms, with 860 degrees of torsion and 15 hours of symptoms leading to a 50% probability of non-salvage. Traditionally, significant ischemic damage to the testis is believed to occur after four to eight hours. Patients presenting after eight hours should still undergo surgical exploration because the viability of testis is difficult to predict. Twenty-five percent of testis undergo atrophy after orchidopexy due to reperfusion injury, which may have delayed and lasting effects on testis viability. Approximately, 50% of men with a history of torsion will have adverse effects on spermatogenesis. As torsion is a traumatic event that disrupts the blood-testis barrier, up to 11% of men will develop anti-sperm antibodies after torsion [13].

Family physicians, who may be the first level of contact with the patient, should have a high index of suspicion to avoid delay in diagnosis and appropriate management. Children presenting with abdominal pain, especially in cold weather should have their testicles examined to rule out torsion [14]. Early scrotal exploration should be considered, once there is a possibility of torsion, to avoid misdiagnosis and loss of testis. In selected cases, even delayed presentation can be given a chance of surgical exploration to salvage the affected testis.

## Conclusions

Testicular torsion is one of the leading causes of acute scrotum presenting in the emergency room. A high degree of clinical suspicion is essential to timely diagnosis and proper management. Scrotal Doppler helps to establish a clinical diagnosis and predict testicular viability on the basis of parenchymal echotexture of the torsed testis. Surgical exploration is the treatment of choice. Patients who present late may still have viable testis
